# Supplementary material for: Meta-Analysis and Gene Set Enrichment Relative to ER Status Reveal Elevated Activity of MYC and E2F in the “Basal” Breast Cancer Subgroup
Source: PLoS One. 2009 Mar 9;4(3):e4710. doi: 10.1371/journal.pone.0004710 (PMC2650420; doi:10.1371/journal.pone.0004710)
Supplement: Dataset S1 — GSEA results from first screen. The file contains a zipped archive of the HTML results of the second GSEA screen. Reports for enriched or depleted datasets may be accessed from the “index.html” link within the my_analysis.GseaPreranked.1219975950625.rpt.zip ZIP archive. Enrichment plots are available for each of the 50 most enriched and depleted gene sets. (13.32 MB ZIP) [file pone.0004710.s001.zip › my_analysis.GseaPreranked.1219975950625.rpt.zip!//GTTNYYNNGGTNA_UNKNOWN.html]

Details for gene set GTTNYYNNGGTNA\_UNKNOWN[GSEA]

|  || Dataset | zSco.ERneg.to.ERpos\_collapsed |
| Phenotype | NoPhenotypeAvailable |
| Upregulated in class | na\_neg |
| GeneSet | GTTNYYNNGGTNA\_UNKNOWN |
| Enrichment Score (ES) | -0.41188875 |
| Normalized Enrichment Score (NES) | -1.6740831 |
| Nominal p-value | 0.009803922 |
| FDR q-value | 0.07515268 |
| FWER p-Value | 0.988 |
Table: GSEA Results Summary

  

Fig 1: Enrichment plot: GTTNYYNNGGTNA\_UNKNOWN      
 Profile of the Running ES Score & Positions of GeneSet Members on the Rank Ordered List

  

| PROBE | GENE SYMBOL | GENE\_TITLE | RANK IN GENE LIST | RANK METRIC SCORE | RUNNING ES | CORE ENRICHMENT || 1 | FGF9 |  |  | 793 | 3.787 | -0.0159 | No |
| 2 | RGS6 |  |  | 2880 | 1.997 | -0.1501 | No |
| 3 | MBNL2 |  |  | 3321 | 1.785 | -0.1626 | No |
| 4 | SLCO3A1 |  |  | 3563 | 1.679 | -0.1613 | No |
| 5 | CAMK2A |  |  | 3621 | 1.651 | -0.1465 | No |
| 6 | WRN |  |  | 3754 | 1.604 | -0.1379 | No |
| 7 | SLC9A7 |  |  | 3790 | 1.589 | -0.1221 | No |
| 8 | JMJD1A |  |  | 3938 | 1.539 | -0.1154 | No |
| 9 | DNAJA1 |  |  | 4099 | 1.474 | -0.1104 | No |
| 10 | PTMS |  |  | 4428 | 1.344 | -0.1195 | No |
| 11 | TUB |  |  | 4500 | 1.318 | -0.1096 | No |
| 12 | NUDC |  |  | 4541 | 1.306 | -0.0975 | No |
| 13 | ZSWIM1 |  |  | 4848 | 1.206 | -0.1067 | No |
| 14 | PIM2 |  |  | 5147 | 1.099 | -0.1164 | No |
| 15 | RFX1 |  |  | 5214 | 1.077 | -0.1089 | No |
| 16 | HPCAL1 |  |  | 5351 | 1.026 | -0.1073 | No |
| 17 | NR4A3 |  |  | 5467 | 0.986 | -0.1045 | No |
| 18 | CHD4 |  |  | 5642 | 0.932 | -0.1069 | No |
| 19 | TAIP-2 |  |  | 5645 | 0.931 | -0.0962 | No |
| 20 | TRERF1 |  |  | 6283 | 0.720 | -0.1359 | No |
| 21 | KIAA1008 |  |  | 6765 | 0.565 | -0.1656 | No |
| 22 | RREB1 |  |  | 6829 | 0.542 | -0.1641 | No |
| 23 | NOS1 |  |  | 7097 | 0.451 | -0.1790 | No |
| 24 | HNRPF |  |  | 7236 | 0.407 | -0.1847 | No |
| 25 | STRN4 |  |  | 7302 | 0.383 | -0.1852 | No |
| 26 | PURG |  |  | 7741 | 0.217 | -0.2157 | No |
| 27 | MAPRE3 |  |  | 7749 | 0.215 | -0.2137 | No |
| 28 | RAB36 |  |  | 7949 | 0.122 | -0.2273 | No |
| 29 | P2RY10 |  |  | 7998 | 0.105 | -0.2297 | No |
| 30 | NR4A1 |  |  | 8011 | 0.101 | -0.2295 | No |
| 31 | OSM |  |  | 8108 | 0.069 | -0.2359 | No |
| 32 | MGAT4B |  |  | 8410 | -0.042 | -0.2581 | No |
| 33 | FLJ14803 |  |  | 8459 | -0.059 | -0.2610 | No |
| 34 | FKRP |  |  | 8618 | -0.137 | -0.2714 | No |
| 35 | KCNK12 |  |  | 8633 | -0.144 | -0.2707 | No |
| 36 | ITGB3BP |  |  | 8803 | -0.209 | -0.2811 | No |
| 37 | TSGA10 |  |  | 9607 | -0.621 | -0.3344 | No |
| 38 | MID2 |  |  | 9677 | -0.659 | -0.3320 | No |
| 39 | OSBPL2 |  |  | 9784 | -0.714 | -0.3317 | No |
| 40 | RAB28 |  |  | 10517 | -1.176 | -0.3733 | No |
| 41 | C13ORF24 |  |  | 10599 | -1.243 | -0.3650 | No |
| 42 | FBXL2 |  |  | 11222 | -1.760 | -0.3915 | Yes |
| 43 | KIFAP3 |  |  | 11242 | -1.780 | -0.3723 | Yes |
| 44 | DCTN3 |  |  | 11354 | -1.885 | -0.3589 | Yes |
| 45 | CAMK1 |  |  | 11753 | -2.268 | -0.3626 | Yes |
| 46 | UNG2 |  |  | 11947 | -2.493 | -0.3483 | Yes |
| 47 | RARA |  |  | 11993 | -2.552 | -0.3222 | Yes |
| 48 | C10ORF57 |  |  | 12211 | -2.852 | -0.3055 | Yes |
| 49 | TBC1D16 |  |  | 12259 | -2.923 | -0.2752 | Yes |
| 50 | STAT6 |  |  | 12446 | -3.173 | -0.2525 | Yes |
| 51 | PCF11 |  |  | 12637 | -3.505 | -0.2262 | Yes |
| 52 | SFRS12 |  |  | 12641 | -3.513 | -0.1858 | Yes |
| 53 | GTF3C1 |  |  | 12854 | -4.018 | -0.1553 | Yes |
| 54 | ARFIP1 |  |  | 12948 | -4.315 | -0.1123 | Yes |
| 55 | OSR2 |  |  | 13056 | -4.668 | -0.0664 | Yes |
| 56 | LRP10 |  |  | 13314 | -7.445 | 0.0005 | Yes |
Table: GSEA details [plain text format]

  

Fig 2: GTTNYYNNGGTNA\_UNKNOWN: Random ES distribution      
 Gene set null distribution of ES for **GTTNYYNNGGTNA\_UNKNOWN**
